# Self-organization and multi-line transport of human spermatozoa in rectangular microchannels due to cell-cell interactions

**DOI:** 10.1038/s41598-020-66803-2

**Published:** 2020-06-17

**Authors:** A. Bukatin, P. Denissenko, V. Kantsler

**Affiliations:** 1 Alferov Saint Petersburg National Research Academic University of the Russian Academy of Sciences, Saint Petersburg, Russia; 2 School of Engeneering, University of Warwick, Coventry, UK; 3 Department of Physics, University of Warwick, Coventry, UK

**Keywords:** Biophysics, Motility, Biomedical engineering, Microfluidics

## Abstract

The journey of sperm navigation towards ovum is one of the most important questions in mammalian fertilisation and reproduction. However, we know very little about spermatozoa propagation in a complex fluidic, chemical and topographic environment of a fertility tract. Using microfluidics techniques, we investigate the influence of cell-cell interactions on spermatozoa swimming behavior in constrained environment at different concentrations. Our study shows that at high enough cell concentration the interaction between boundary-following cells leads to formation of areas with preferential direction of cell swimming. In the microchannel of a rectangular cross-section, this leads to formation of a “four-lane” swimming pattern with the asymmetry of the cell distribution of up to 40%. We propose that this is caused by the combination of cell-cell collisions in the corners of the microchannel and the existence of morphologically different spermatozoa: slightly asymmetric cells with trajectories curved left and the symmetric ones, with trajectories curved right. Our findings suggest that cell-cell interactions in highly folded environment of mammalian reproductive tract are important for spermatozoa swimming behavior and play role in selection of highly motile cells.

## Introduction

To fertilize an egg, mammalian sperm must travel thousands of its body lengths in the reproductive tract. While passing the cervix, uterus and oviduct, spermatozoon encounters different geometric structures, viscosity, pH, and temperature^1,2^. Over the course of evolution, spermatozoa of different species have developed a whole range of mechanisms and strategies to pass the obstacles and reach the egg^3,4^. These mechanisms include surface following behavior^5^, rheotaxis^6,7^, chemotaxis^8,9^, and thermotaxis^10,11^.

Motility is one of the key features of spermatrozoa that allows for successful fertilization. Spermatozoa motility is provided by the flagellum periodical beating which, coupled with elastic flagellum-fluid interaction, makes the flow around the cell irreversible thus generating cell propulsion. To balance the moment of forces occurring due to flagellum motion, the cell head rotates^12,13^. Depending on the environmental conditions (temperature, viscosity) cells can swim in a variety of hydrodynamic modes that correspond to different types of three-dimensional trajectories: typical, helical, hyper-helical, hyper-activated or chiral ribbons^14,15^, and exhibit different patterns of flagellum beating^16^. Recently, different patterns of sperm behavior were observed in mice using *in vivo* optical coherence tomography^17^.

Female reproduction tract has a complex 3-dimentional structure with large surface-to-volume ratio covered with cilia^2,18,19^. Between the secondary folds of mucosa of the tract, sperm can be stored for many hours waiting for ovulation^20^. Recent experiments on spermatozoa of various types of mammals and bacteria, as well as numerical simulations show that hydrodynamic interaction of cells with a solid boundary leads to their surface accumulation^21–25^. In most cases, the flagellum-beating pattern near a surface remains three-dimensional with helical structure^26^. Despite the fact that for all human sperm cells, the direction of flagellum beating plane rotation seen from head-on is counter clockwise, there are two kinematically distinct swimming states differing in the cells’ turning direction against the fluid flow due to rheotactic behavior^27^. The cell turning direction strongly correlates with the angle between the midpiece and the head of the cell. Moreover, cells located at a distance of less than 1 μm from the surface can switch into a two-dimensional “slither” swimming mode, which is distinguished by the planar flagellum beating in the boundary plane^28^. For human sperm, such a swimming mode can only occur if viscosity of the medium is more than 20 mPa*s. In the presence of multi-scale surfaces, cells can concentrate near structures with lower dimensions. In rectangular microchannels, they concentrate in the corners due to interaction with the walls^29,30^. By designing channels of a particular geometry, it is possible to control the swimming direction hence arranging cell flows^5^.

The ability of spermatozoa to move along the surface boundaries, as well as against the fluid flow, correlates with a low level of DNA fragmentation^31,32^. Nowadays, this phenomena is being used for development of microfluidic devices for cell selection for IVF or ICSI procedure^33–35^, as it has been shown that low DNA fragmentation and high swimming velocity of sperm cells correlate with success of IVF and ICSI^36,37^. Recent studies show that selection of motile spermatozoa in the sorting microfluidic devices displays significant advantages compared to traditional methods of swim-up and centrifugation in a density gradient^38–40^.

Due to the fact that millions of cells are injected during coitus into a female reproduction tract, spermatozoa rarely swim alone. Thus, it is important to study cell-cell interactions and their influence on the navigation process. The fluid velocity field created by a sperm cell in bulk or near a wall is similar to that generated by a bacteria E.coli and can be described by the force dipole approximation with a good accuracy^41,42^. Superposition of fluid flows induced by two cells cause hydrodynamic dipole-dipole interaction between them. At high concentrations, this leads to synchronization of the flagella beating and the emergence of collective behavior^43–45^. In case of sea urchins spermatozoa, cell-cell interaction leads to formation of self-organized vortex arrays^46^, and in case of ram spermatozoa and E.coli – to formation of self-organized direct flows in microchannels^47^. Nevertheless, it is not completely known how the presence of different types of kinematic swimming modes of the cells, characterized by different types of trajectories and flagella beating patterns, affects cell-cell interactions and navigation in a female reproduction tract. Understanding the influence of cells’ kinematic states on collective behavior is difficult due to high concentration of cells when such behavior starts playing significant role^48,49^.

In this work, we used sperm accumulation in the corners of rectangular microchannels^29^ to study cell-cell interactions. The total concentration of cells in the channels was relatively low, thus it was possible to track individual cells on the lower wall (the wall closer to the objective of an inverted microscope) and divide them into two kinematic states, which are cells turning left and cells turning right if observed from a direction normal to the wall. Our results show that cells moving in opposite directions organize into “four-lane” flows with similar structure, which has been universally observed in all microchannels connecting sperm-filled chambers. This self-organisation strongly depends on cell concentration and is caused by cell-cell interactions that lead to cells switching from one kinematic state to another.

## Results

**Figure 1:**
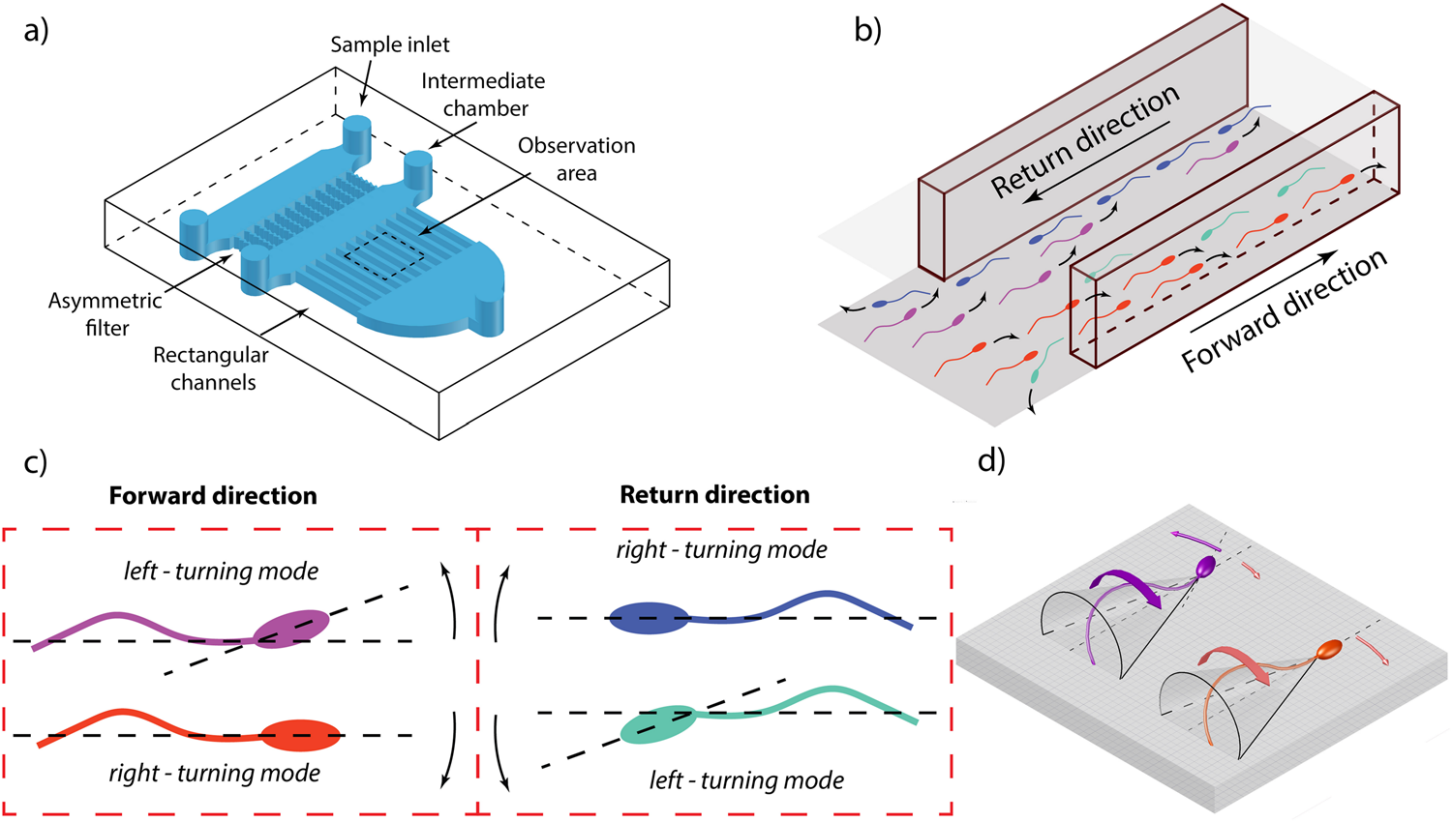
Microfluidic device for quantitave investigation of cell-cell interactions. (**a**) Schematic view of the device: cells from the sample chamber are guided into the intermediate chamber by one-way channels and then get into rectangular microchannels. Spermatozoa observation was performed simultaneously at bottom walls of the 4 channels by inverted brightfield microscope with 20×/0.75 objective, creating a field of view of 850×675 μm; (**b**) schematic view of cells at the entrance of a channel: cells get into channels from top and bottom planes and start forming the “four-lane” pattern; (**c**) different swimming modes of spermatozoa in the channels coded in color; (**d**) schematic view of differences between right – turning and left – turning behavior: in right – turning mode the average angle between the midpiece and the head of the cell is close to zero, however in left – turning mode this angle has a notable value^27^.

To study spermatozoa cell-cell interactions, we have developed a microfluidic device that contains 16 rectangular microchannels with a length of 5 mm (Figs. 1a and S1). Microchannels connect two chambers, into one of which motile cells from the seminal fluid are guided using asymmetric one-way microchannels proposed earlier^5^. The inlets and outlets of the device were sealed hence there were no fluid flows in the channels. After 15–20 minutes the experiment started, the concentration of motile cells in the chambers on both sides of the microchannels was equalized and the total number of cells moving in both directions in the channels became similar. Due to the fact that sperm cells accumulate on surfaces^26^ they enter the microchannels mostly from the upper and lower walls of the chambers (Fig. 1b). When in the channels, spermatozoa continue to swim along the upper and lower walls concentrating in the corners with the cell concentration on the sidewalls and in the bulk close to zero. To confirm that the number of cells in the bulk and on the sidewalls is low, we calculated the number of the out-of-focus cells near the bottom walls of the channels. In all our experiments the number of such cells was 11–25% (Fig. S2). Using inverted brightfield microscope with low magnification and high numeric aperture objective (20×/0.75), we simultaneously observe sperm cells near the bottom walls of four channels. The field of view was 850×675 μm located in the middle of the 5 mm channels’ span. The width of the microchannels was chosen comparable to the length of human sperm at 60 μm and the height of the microchannels was 100 μm. All the measurements were conducted at the bottom wall of the channels. We have visually observed that the behaviour of the cells at the upper wall has been symmetric to that at the lower wall where the measurements have been conducted. From this point we will be referring to the channels as two-dimensional, keeping in mind that the observations are constrained to the lower wall of the rectangular cross section.

When displaying rheotactic behavior, human sperm cells have been shown to swim in two kinematically distinct modes differing in their turning direction^27^. As these modes strongly correlate with the angle between cell’s mid-piece and the head, we assume that they also exist in the absence of any fluid flows and determine cells’ turning direction when following a flat wall. Classification of all types of cells in the microchannels (swimming forward and return with right-turning and left-turning behavior) is coded in cells’ color and is shown in Fig. 1c,d. To quantitatively analyze cell-cell scattering behavior on each frame in each channel, we have calculated the linear concentration **n** of cells per unit length of a microchannel. Cell concentration has been non-dimensionalized by the inverse cell length **n**_**0**_, i.e. the concentration at which cells can be placed in corners at equal spacing without intersecting. Statistics of cell swimming directions was separately calculated in six equal ranges of cell concentration. Since the length of a human spermatozoon is about 60 μm (5 μm head and 55 μm flagellum)^1^, the base concentration of cells in the microchannels **n**_**0**_ = 33 cells/mm. To assess the degree of asymmetry in the distribution of cells an enrichment index ${R}_{{eff}}$ was introduced:

$${R}_{{eff}}=\frac{|{P}_{2}-{P}_{1}|}{{P}_{2}+{P}_{1}}\ast 100 \% $$

**Figure 2:**
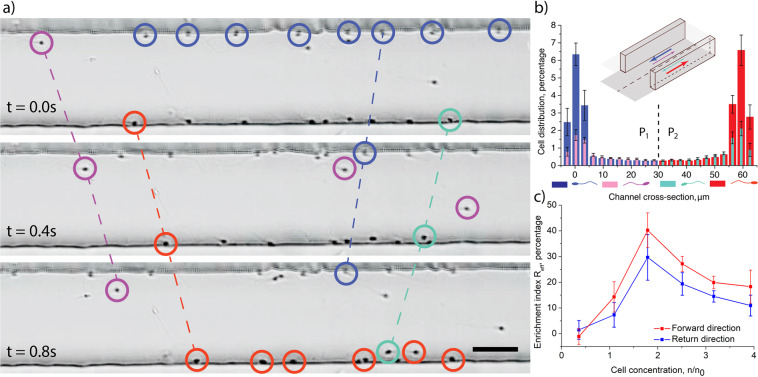
Cells distribution on a bottom wall of a rectangular microchannel indicates «four-lane» self-organization pattern. (**a**) Three time-lapse images showing that human sperm cells prefer to swim in the right corner of a rectangular microchannel and form chains that move in one direction, circle’s color indicates cell’s swimming mode, scale bar is 30 μm; (**b**) histograms of sperm cells average distribution across the channels during 6 experiments where cell concentration was **n** ~ 1.8*n_**0**_, colors of the bars indicate the swimming modes, error bars - standard errors; (**c**) enrichment index ${R}_{{eff}}$ as a function of cell concentration for cells swimming in forward and return directions indicates the difference between the number of cells in two halves of the channels, each point represents at least 3 experiments where more than 918 individual trajectories in four channels were analyzed, error bars indicate standard errors.

${P}_{1}$ and ${P}_{2}$ are the probabilities of finding cells in the half of the channel close to the right or left corner according to the forward direction as indicated in Fig. 2b. The enrichment index was calculated for cells swimming in forward and return directions separately. To get the dependence of the enrichment index on cell concentration, the total of 41 experiments with samples obtained from two healthy donors have been performed. For each concentration, at least three experiments were made and in each channel at least 918 individual cell trajectories have been analyzed. From all the data, only those channels were analyzed where the average number of cells in two halves of the channels differs by less than 10%. Higher difference indicates that the numbers of cells swimming in the opposite directions are not similar which may affect the swimming pattern.

Cell-cell interactions in the corners of the channels lead to the formation of a specific pattern of cell distribution (Fig. 2, supplementary video 1–3). Cells prefer to swim in the right corner of the channel relatively to their swimming direction. Similar behavior was reported previously for E.coli bacteria^50^. This happens because bacterial cells swim in a clockwise manner and only exhibit the right-turning mode. On contrast, different sperm cells display both left-turning and right-turning behavior. At low concentrations, when **n** < **n**_**0**_ the cells do not have a preferred swimming pattern and occupy corners evenly. This is in a good agreement with the previous study^27^ where we have shown that the number of left-turning and right-turning cells is close. However, at higher concentrations cells start organizing a well-defined direction of motion in each corner and form a specific pattern of cell distribution (Figs. 2b,c and S3). This pattern is the same in all the channels for cells from different donors. The effect is the most pronounced at cell concentration n = 1.8 * n_**0**_ when the enrichment index is R_eff_ = 35–40% depending on the direction of cell motion. At higher concentrations of cells, the enrichment index decreases up to 15–20% when the concentration becomes n = 3.9 * n_**0**_. Enrichment indices for different directions slightly differ due to longer travelling way to the observation point for the returning cells. The slight change in the enrichment index does not affect the organization of cell flows.

**Figure 3:**
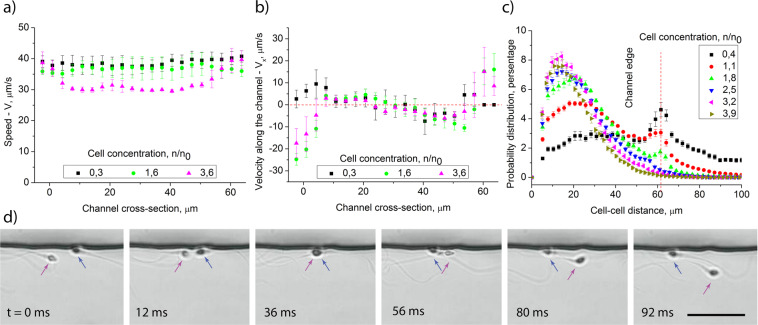
Properties of the «four-lane» swimming pattern. (**a**) Spermatozoa average speed across the channels indicate that at high concentrations less motile cells are displaced from the corners; (**b**) average velocity along the channels specify the «four-lane» pattern: cells in each line swim in the opposite direction to the neighbor lines; (**c**) distribution of cell-cell distance at different concentrations; (**d**) six time lapse images showing an example of a scattering event, the scale bar is 30 μm. Error bars indicate standard errors.

To study the characteristics of the cells’ swimming pattern at different concentrations, we have analyzed their average velocity across the channel and its projection on the channel’s axis (Figs. 3a,b and S4). At low concentrations, the cell speed across the channels is uniform, however, as cell concentration increases, the speed in the center of the channel decreases. It shows that less motile cells are displaced from the corners of the channels due to cell-cell interactions. This fact is in agreement with the previous study where it was shown that cells moving in the corners of the microchannels have less DNA fragmentation and therefore are more fertile^31^. The projection of average sperm velocity on the channel’s axis shows in more details the structure of the cell-moving pattern. It can be named “four-lane” traffic resembling a four-lane road, where the direction of motion in a specific line is carried out in the opposite direction to the neighbor lines (see Fig. 3b). According to this pattern the symmetric cells with the flagellum attached to the cell body along the axis, mainly move in the opposite corners of the channels. The asymmetric cells with the flagellum attached to the head at an angle, move along the sidewall just off these corners in the opposite direction as illustrated in Fig. 1b. This “four-lane” pattern only appears when the number of cells is enough for effective cell-cell scattering and is not formed at low concentrations when cells are not interacting.

To understand why such swimming behavior appears, we have grouped all cells into pairs of nearest neighbors and calculated distributions of distances between their heads (Fig. 3c). The distribution strongly depends on the cell concentration and has two maxima. The first one is in the range 13–32 μm depending on the concentration while the second one is about 60 μm, which is close to the channel’s width. At low concentrations, maximum at 60 μm dominates but at higher concentrations maximum at 13–32 μm prevails. To figure out the meaning of such cell distributions, we note that when the number of cells is low, they swim mostly individually in the corners. Thus, the maximum of the distribution function at 60 μm corresponds to the distance between cells swimming in different corners of a channel. At higher concentrations, the cells start interacting with each other in the corners and form chains moving in one direction (Fig. 2a). Therefore, the maximum at 13–32 μm corresponds to the most probable distance between cells swimming in the same corner of a channel. The distance between cells in a corner is determined by cell-cell and cell-surface interactions. These interactions could be carried out by hydrodynamic and contact forces^41,51^. According to the “four-lane” flow pattern and the distribution of cells in channels (Fig. 2) we suggest that spermatozoa moving in corners in the same direction mainly interact hydrodynamically although cells moving in opposite directions scatter by contact collisions (Fig. 3d). To consider the influence of hydrodynamic forces on cell-cell distance, we should take into account flow fields generated by sperm cells. The structure of these flow fields is similar to flow fields generated by other pusher microorganisms such as E.coli and can be described by a simple force dipole model^41,42^. Numerical simulations show that near a corner there is a potential well for pushers that induce their accumulation in corners^30^. For cells that swim head-to-tail in a corner, hydrodynamic interaction is repulsive, therefore it causes a formation of cell trains moving in one direction. At high concentrations **n** $\ge$ 2.5*n_**0**_ the optimal distance between the cells saturates at 12–15 μm. Such behavior can be explained by a repulsive force that exceeds corner attractive force and pushes less motile cells out from the corner. This leads to a decrease of the enrichment index (Fig. 2c) and average cells speed in the center of a channel at high cell concentration (Fig. 3a). Moreover, this repulsive force pushes the cells from the bottom wall to the bulk and sidewalls of the channels, which leads to an increment of the number of observed out-of-focus cells (Fig. S2).

**Figure 4:**
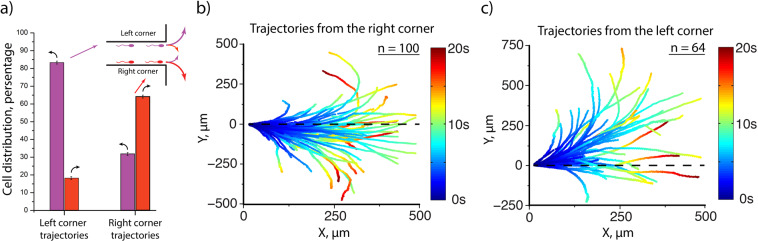
Individual trajectories of sperm cells after the end of a channel. (**a**) Distribution of cells turning behavior when leaving the channels indicates that cells in the right corner more often have the turning direction, typical for the left corner. Distributions are based on three experiments, error bars indicate standard errors and are less than 1%; (**b**) trajectories of the cells leaving the right corner of a channel; (**c**) trajectories of the cells leaving the left corner of a channel.

Self-organized spermatozoa swimming pattern indicates that due to cell-cell interactions, the cells with left-turning behavior can change the turning direction and integrate into a stream of right-turning cells. Such changes of turning behavior may be caused by nonlinear instability in flagellum dynamics^52^. To investigate the stability of this transition we have captured and analyzed trajectories of individual sperm cells after the end of the channels at the cell concentration n ~ 1.3*n_**0**_ in three experiments (Fig. 4). It turned out that 30% and 17% of cells swimming in the right and left corners respectively change their turning direction after the end of the channels (Fig. 4a). This shows that left-turning cells are less stable in the corners and leads to increasing the number of cells, swimming in the right corner and to formation of observed asymmetric pattern of the cell flows. After passing the channel and getting into the chamber, some of the cells from the main flow in a right corner display their original (left) turning direction (Fig. 4b). Smaller number of cells that moved in the left corner display the right turning direction (Fig. 4c). This confirms that the position of the left-turning cells in the corners is less stable. On the contrary, cells with right-turning behavior are more stable and rarely swim in left corners.

## Conclusions

We have quantitatively investigated human spermatozoa swimming behavior on the bottom walls and in the corners of rectangular microchannels as a function of cell concentration. Our results demonstrate that while the total number of cells swimming in the forward and return directions is similar, when the number of cells exceeds a certain value they start forming oppositely directed cell flows in the corners of a channel and a “four-lane” swimming pattern on the walls. In all the channels cells prefer to swim in the right corner according to the swimming direction. Self-organization of such swimming pattern occurs due to cell-cell interactions in the corners of a channel. At low concentrations, when the cells swim mostly independently, the number of cells in the right and left corners of the channels is similar. When the concentration increases, the asymmetric flow pattern begins to appear. Maximum asymmetry occurs at the cell concentration n = 1.8*n_**0**_ when the most probable distance between the heads of the nearest cells becomes 20 μm, a third of their length. At such intercellular distances, human spermatozoa overlap and start repelling hydrodynamically. The asymmetric left-turning cells are being pushed out of the corner more frequently than the right-turning symmetric cells thus leaving the symmetric cells to define the mean direction of motion in the corners. When the concentration of the cells increases further, the distance between cells’ heads decreases to 12–15 μm so that all types of cells leave the corners, which leads to reduction in the enrichment index and formation of the less pronounced flow pattern.

In the contest of natural reproduction, our results show the importance of cell-cell interactions for spermatozoa swimming behavior. In the highly folded epithelium of a reproductive tract, such interactions may become an additional instrument for selection of highly motile spermatozoa as less motile asymmetrically-shaped cells can be pushed away from the surface by such interactions. These rejected cells might have high DNA fragmentation^31^ and move with a lower speed, having lower chances to reach and fertilize the egg.

## Materials and methods

### Device fabrication

Microfluidic devices were made using standard soft-lithography process. The single-layer mold was produced using SU-8 2075 (MicroChem Corporation) on a silicone wafer by optical contact lithography with a plastic mask. The microfluidic chip was cast from polydimethylsiloxane (PDMS) (Sylgard 184; Dow Corning). The PDMS prepolymer and the curing agent were mixed in a ratio of 10:1 w/w, degassed, poured into the mold and cured at 65 C for 4 h in an oven. After that, PDMS replica was detached from the mold, inlet and outlet holes were punched and it was bonded with a cover glass slide after oxygen plasma treatment. Before the experiment the channels were filled with medium. After sample injection the inlets were sealed by metal stubs thus there were no fluid flows in the channels of the device.

### Sample preparation

We used the sample preparation protocol similar to our previous work^27^. The medium for filling the microfluidic devices was based on a standard Earle’s Balanced Salt Solution, which is commonly used for IVF procedures^53^. It contains 66.4 mM NaCl, 5.4 mM KCl, 1.6 mM CaCl_2_, 0.8 mM MgSO_4_, 1 mM N_2_H_2_PO_4_, 26 mM NaHCO_3_, 5.5 mM D-Glucose with pH 7.1–7.8 supplemented with 2.5 mM Na pyruvate as an energy source for sperm cells^54^ and 0.2% wt serum albumin to prevent sticking of cells on the channel walls.

Human sperm samples from two healthy undisclosed normozoospermic donors were obtained from the Urology Clinic of the First Pavlov Medical University of St. Petersburg. Donors provided informed consent in accordance with the regulations of the Ethical Committee of St. Petersburg Academic University, which granted approval to this research under record number 04/14. The ejaculated samples were incubated at 37 °C for 1 h before injection into the sample chamber of the device. Motile cells fill two chambers and the experimental channels between them within 10–20 min by passing the one-way filtration channels.

### Microscopy and experimental procedure

To analyze the swimming pattern of sperm cells moving in the opposite directions, we used a Nikon TE2000U inverted microscope with brightfield illumination (20×, NA 0.75 objective, Edgertronic SC1 camera at 50 fps, 5000 frames). The field of view (normally 850×675 μm) was selected in the central part of the channels and contained four identical channels on each frame. The focal plane was set slightly above the bottom wall of the channels, thus all the cells on the wall were seen as dark spots. The cells that were above the focal plane were seen as bright spots. The individual trajectories of spermatozoa on the captured videos were reconstructed by applying a custom-made particle-tracking algorithm. Cell recognition was performed by an algorithm described in^55^. Detailed videos of cell-cell scattering events were captured with an 40×/NA 1.3 objective and Edgertronic SC1 camera (500 fps).

## Supplementary information

Supplementary Information.

Supplementary Information 2.

Supplementary Information 3.

Supplementary Information 4.

## References

[1] Gaffney EA, Gadelha H, Smith DJ, Blake JR, Kirkman-Brown JC (2011). Mammalian Sperm Motility: Observation and Theory. Annu. Rev. Fluid Mech..

[2] Suarez SS, Pacey AA (2006). Sperm transport in the female reproductive tract. Human Reproduction Update.

[3] Simmons LW, Fitzpatrick JL (2012). Sperm wars and the evolution of male fertility. Reproduction.

[4] Tourmente M, Gomendio M, Roldan ERS (2011). Sperm competition and the evolution of sperm design in mammals. BMC Evolutionary Biology.

[5] Denissenko P, Kantsler V, Smith DJ, Kirkman-Brown J (2012). Human spermatozoa migration in microchannels reveals boundary-following navigation. PNAS.

[6] Brethertron FP, Rothschild FRS (1961). Rheotaxis of spermatozoa. Proc. R. Soc. Lond. B.

[7] Kantsler V, Dunkel J, Blayney M, Goldstein RE (2014). Rheotaxis facilitates upstream navigation of mammalian sperm cells. eLife.

[8] Eisenbach, M. & Giojalas, L. C. Sperm guidance in mammals — an unpaved road to the egg. Nature reviews, molecular cell biology, 2006, vol. 7 10.1038/nrm1893 16607290

[9] Yoshida, M. & Yoshida, K. Sperm chemotaxis and regulation of flagellar movement by Ca^2+^. Molecular Human Reproduction, Volume 17, Issue 8, August 2011, Pages 457–465 10.1093/molehr/gar041 21610215

[10] Bahat, A. *et al*. Thermotaxis of mammalian sperm cells: A potential navigation mechanism in the female genital tract. Nature medicine, vol. 9, No 2, 2003 10.1038/nm0203-149 12563318

[11] Boryshpolets S, Perez-Cerezales S, Eisenbach M (2015). Behavioral mechanism of human sperm in thermotaxis: a role for hyperactivation. Human Reproduction.

[12] Phillips DM (1972). Comparative analysis of mammalian sperm motility. The journal of cell biology.

[13] Ishijima S, Hamaguchi MS, Naruse M, Ishijima SA, Hamaguchi Y (1992). Rotational movement of a spermatozoon around its long axis. J. exp. Biol..

[14] Su, T.-W., Xue, L. & Ozcan, A. High-throughput lensfree 3D tracking of human sperms reveals rare statistics of helical trajectories. PNAS, 2012, vol. 109, no. 40. 10.1073/pnas.1212506109 PMC3479566 22988076

[15] Su T-W (2013). Sperm Trajectories Form Chiral Ribbons. Scientific reports.

[16] Smith DJ (2009). Propagation in the Flagella of Migrating Human Sperm, and Its Modulation by Viscosity. Cell Motility and the Cytoskeleton.

[17] Wang S, Larina IV (2018). *In vivo* three-dimensional tracking of sperm behaviors in the mouse oviduct. Development.

[18] Suarez, S. S. Mammalian Sperm Interactions with the Female Reproductive Tract. *Cell Tissue Res*. 2016 January; 363(1), 185–194 10.1007/s00441-015-2244-2 PMC4703433 26183721

[19] Suarez SS, Brockman K, Lefebvre R (1997). Distribution of Mucus and Sperm in Bovine Oviducts after Artificial Insemination: The Physical Environment of the Oviductal Sperm Reservoir. Biology of reproduction.

[20] Koyama, H., Shi, D. & Fujimori, T. Biophysics in oviduct: Planar cell polarity, *cilia, epithelial fold and tube morphogenesis, egg dynamics. Biophysics and Physicobiology*, Vol. 16 (2019). 10.2142/biophysico.16.0_89 PMC6435019 30923666

[21] Berke AP, Turner L, Berg HC, Lauga E (2008). Hydrodynamic Attraction of Swimming Microorganisms by Surfaces. PRL.

[22] Elgeti J, Kaupp UB, Gompper G (2010). Hydrodynamics of Sperm Cells near Surfaces. Biophysical Journal.

[23] Cosson J, Huitorel P, Gagnon C (2003). How Spermatozoa Come to Be Confined to Surfaces. Cell Motility and the Cytoskeleton.

[24] Smith DJ, Blake JR (2009). Surface accumulation of spermatozoa: a fluid dynamic phenomenon. The Mathematical. Scientist.

[25] Smith DJ, Gaffney EA, Blake JR, Kirkman-Brown JC (2009). Human sperm accumulation near surfaces: a simulation study. J. Fluid Mech..

[26] Woolley DM (2003). Motility of spermatozoa at surfaces. Reproduction.

[27] Bukatin, A., Kukhtevich, I., Stoop, N., Dunkel, J. & Kantsler V. Bimodal rheotactic behavior reflects flagellar beat asymmetry in human sperm cells. *PNAS*, vol. 112, no. 52 (2015). 10.1073/pnas.1515159112 PMC4703022 26655343

[28] Nosrati R, Driouchi A, Yip CM, Sinton D (2015). Two-dimensional slither swimming of sperm within a micrometre of a surface. Nature communications.

[29] Nosrati R, Graham PJ, Liu Q, Sinton D (2016). Predominance of sperm motion in corners. Scientific Reports.

[30] Shum H, Gaffney EA (2015). Hydrodynamic analysis of flagellated bacteria swimming in corners of rectangular channels. Physical review E.

[31] Eamer L (2016). Turning the corner in fertility: high DNA integrity of boundary- following sperm. Lab Chip.

[32] Riordon J (2019). Two-Dimensional Planar Swimming Selects for High DNA Integrity Sperm. Lab Chip.

[33] Nosrati R (2014). Rapid selection of sperm with high DNA integrity. Lab Chip.

[34] Nosrati R (2017). Microfluidics for sperm analysis and selection. Nat Rev Urol..

[35] Weng L (2019). IVF-on-a-Chip: Recent Advances in Microfluidics Technology for *In Vitro* Fertilization. SLAS Technology.

[36] Moore RW, Wilson MC, Duganzich DM (1985). Swimming speed and fertilisation rates of ram sperm from high and low prolificacy populations. Proceedings of the New Zealand Society of Animal Production.

[37] Jin, J. *et al*. Effect of sperm DNA fragmentation on the clinical outcomes for *in vitro* fertilization and intracytoplasmic sperm injection in women with different ovarian reserves. *Fertil Steril*. 2015 Apr;103(4):910-6. 10.1016/j.fertnstert.2015.01.014 25747135

[38] Yetkinel, S. *et al*. Effects of the microfluidic chip technique in sperm selection for intracytoplasmic sperm injection for unexplained infertility: a prospective, randomized controlled trial. *J Assist Reprod Genet*. 2019 Mar;36(3):403-409 10.1007/s10815-018-1375-2 PMC6439003 30542782

[39] Quinn, M.M. *et al*. Microfluidic sorting selects sperm for clinical use with reduced DNA damage compared to density gradient centrifugation with swim-up in split semen samples. *Hum Reprod*. 2018 Aug 1;33(8):1388-1393 10.1093/humrep/dey239 30007319

[40] Nagata MPB (2018). Live births from artificial insemination of microfluidic sorted bovine spermatozoa characterized by trajectories correlated with fertility. PNAS April 3.

[41] Drescher K, Dunkel J, Cisneros LH, Ganguly S, Goldstein RE (2011). Fluid dynamics and noise in bacterial cell**–**cell and cell**–**surface scattering. PNAS.

[42] Ishimoto K, Gadelha H, Gaffney EA, Smith DJ, Kirkman-Brown J (2017). Coarse-Graining the Fluid Flow around a Human Sperm. PRL.

[43] Elgeti J, Winkler RG, Gompper G (2015). Physics of Microswimmers – Single Particle Motion and Collective Behavior. Rep. Prog. Phys..

[44] Yang Y, Elgeti J, Gompper G (2008). Cooperation of sperm in two dimensions: Synchronization, attraction, and aggregation through hydrodynamic interactions. Physical review E.

[45] Tung C (2017). Fluid viscoelasticity promotes collective swimming of sperm. Scientific Reports.

[46] Riedel IH, Kruse K, Howard J (2005). A Self-Organized Vortex Array of Hydrodynamically Entrained Sperm Cells. Science.

[47] Creppy A (2016). Symmetry-breaking phase transitions in highly concentrated semen. J. R. Soc. Interface.

[48] Creppy, A., Praud, O., Druart, X., Kohnke, P.L., Plourabou, F. Turbulence of swarming sperm. Physical Review E, 2015, vol. 92, no. 3. 10.1103/PhysRevE.92.032722 26465513

[49] Wioland H, Lushi E, Goldstein RE (2016). Directed collective motion of bacteria under channel confinement. New J. Phys..

[50] DiLuzio, W. R. *et al*. Escherichia coli swim on the right-hand side. *Nature*. 2005 Jun 30;435(7046):1271-4. 10.1038/nature03660 15988531

[51] Contino M., Lushi, E., Tuval, I., Kantsler, V. & Polin M. Microalgae scatter off solid surfaces by hydrodynamic and contact forces. *Phys Rev Lett*. 2015 Dec 18;115(25):258102. 10.1103/PhysRevLett.115.258102 26722946

[52] Gadelha H, Gaffney EA, Smith DJ, Kirkman-Brown JC (2010). Nonlinear instability in flagellar dynamics: a novel modulation mechanism in sperm migration. J. R. Soc. Interface.

[53] Karamalegos C, Bolton VN (1999). A prospective comparison of ‘in house’ and commercially prepared Earle’s balanced salt solution in human *in-vitro* fertilization. Hum Reprod..

[54] Darr CR (2016). Lactate and Pyruvate Are Major Sources of Energy for Stallion Sperm with Dose Effects on Mitochondrial Function, Motility, and ROS Production. Biol Reprod..

[55] Crocker JC, Grier DG (1996). Methods of Digital Video Microscopy for Colloidal Studies. Journal of Colloid and Interface Science.

