# Supplementary material for: Self-organization and multi-line transport of human spermatozoa in rectangular microchannels due to cell-cell interactions
Source: Sci Rep. 2020 Jun 17;10:9830. doi: 10.1038/s41598-020-66803-2 (PMC7299960; doi:10.1038/s41598-020-66803-2)
Supplement: Supplementary file 1 — Supplementary Information. [file 41598_2020_66803_MOESM1_ESM.docx]

Supplementary information

**Self-organization and multi-line transport of human spermatozoa in rectangular microchannels due to cell-cell interactions.**

A. Bukatin^1,*^, P. Denissenko^2^, V. Kantsler^3^

^1^ Alferov Saint Petersburg National Research Academic University of the Russian Academy of Sciences, Saint Petersburg, Russia

^2^ School of Engeneering, University of Warwick, Coventry, UK.

^3^ Department of Physics, University of Warwick, Coventry, UK.

^*^ Correspondence and requests for materials should be addressed to A.B. (email: antbuk.fiztek@gmail.com)


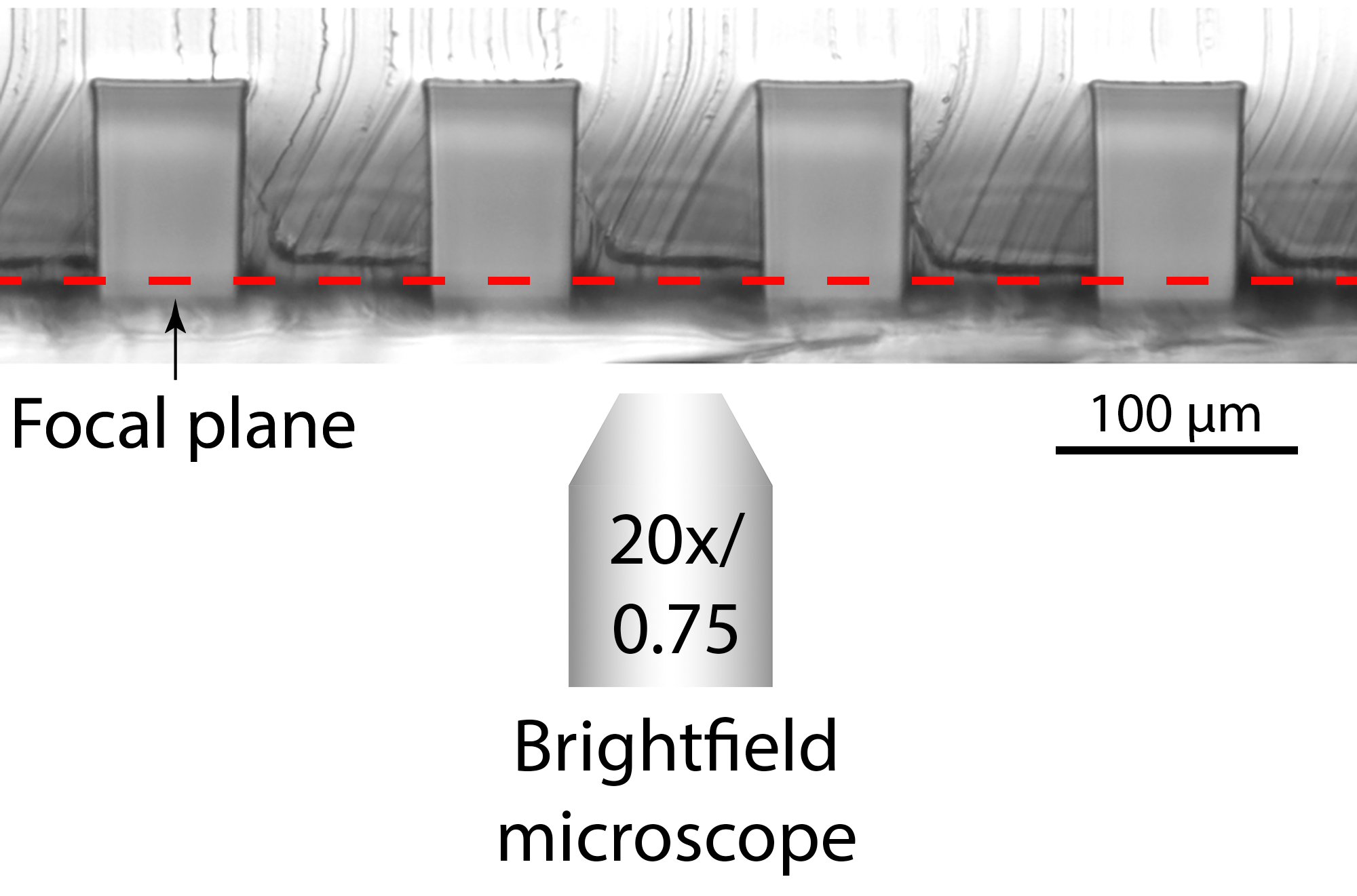


Figure S1. Microchannels cross-section: spermatozoa observation on the bottom wall of the channels was performed by an inverted brightfield microscope with a 20x/0.75 objective lense, field of view was 850x675 μm and captured 4 channels simultaneously.


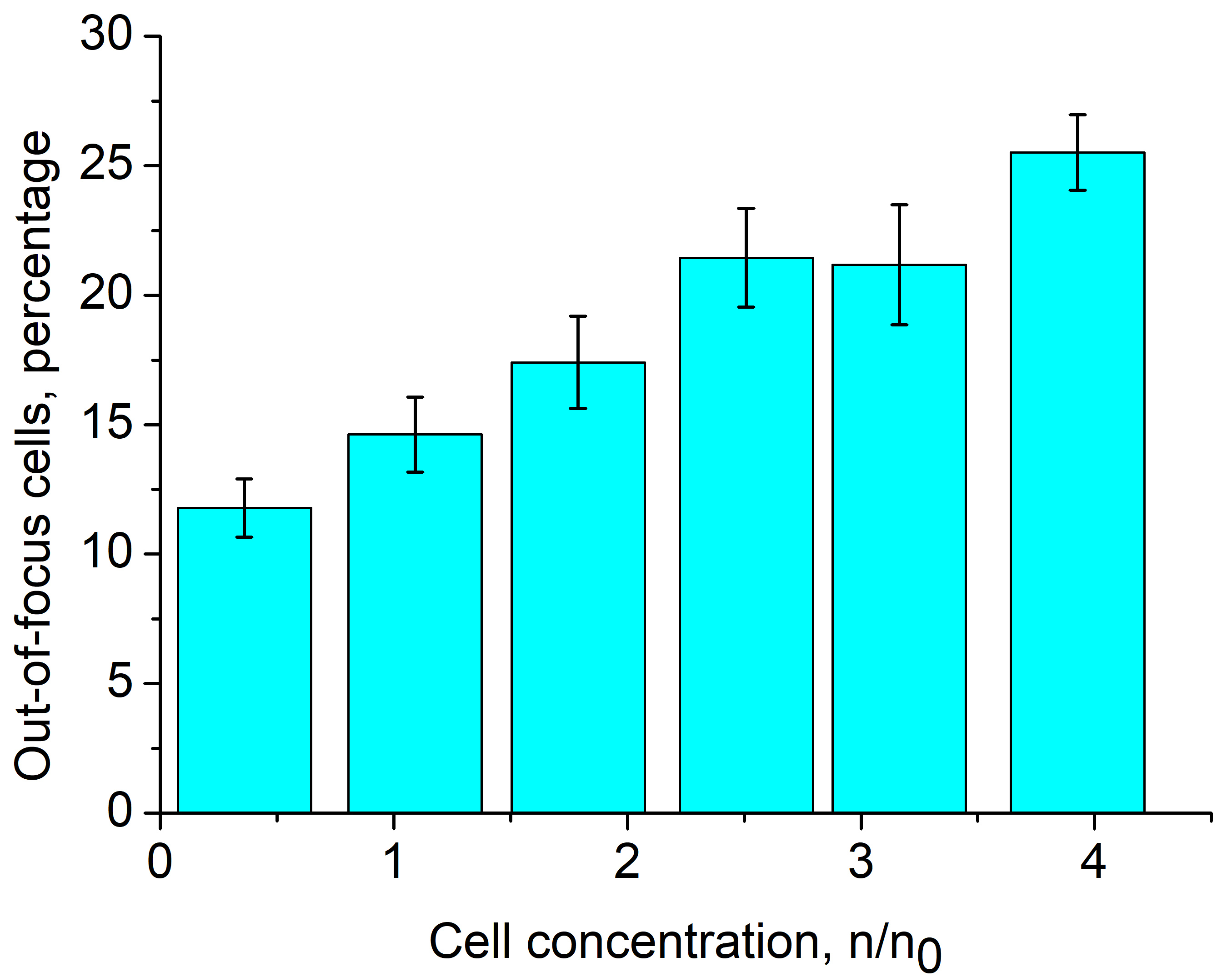


Figure S2. Average number of out-of-focus cells near the bottom walls of the microchannels, error bars indicate standard errors.

| a)  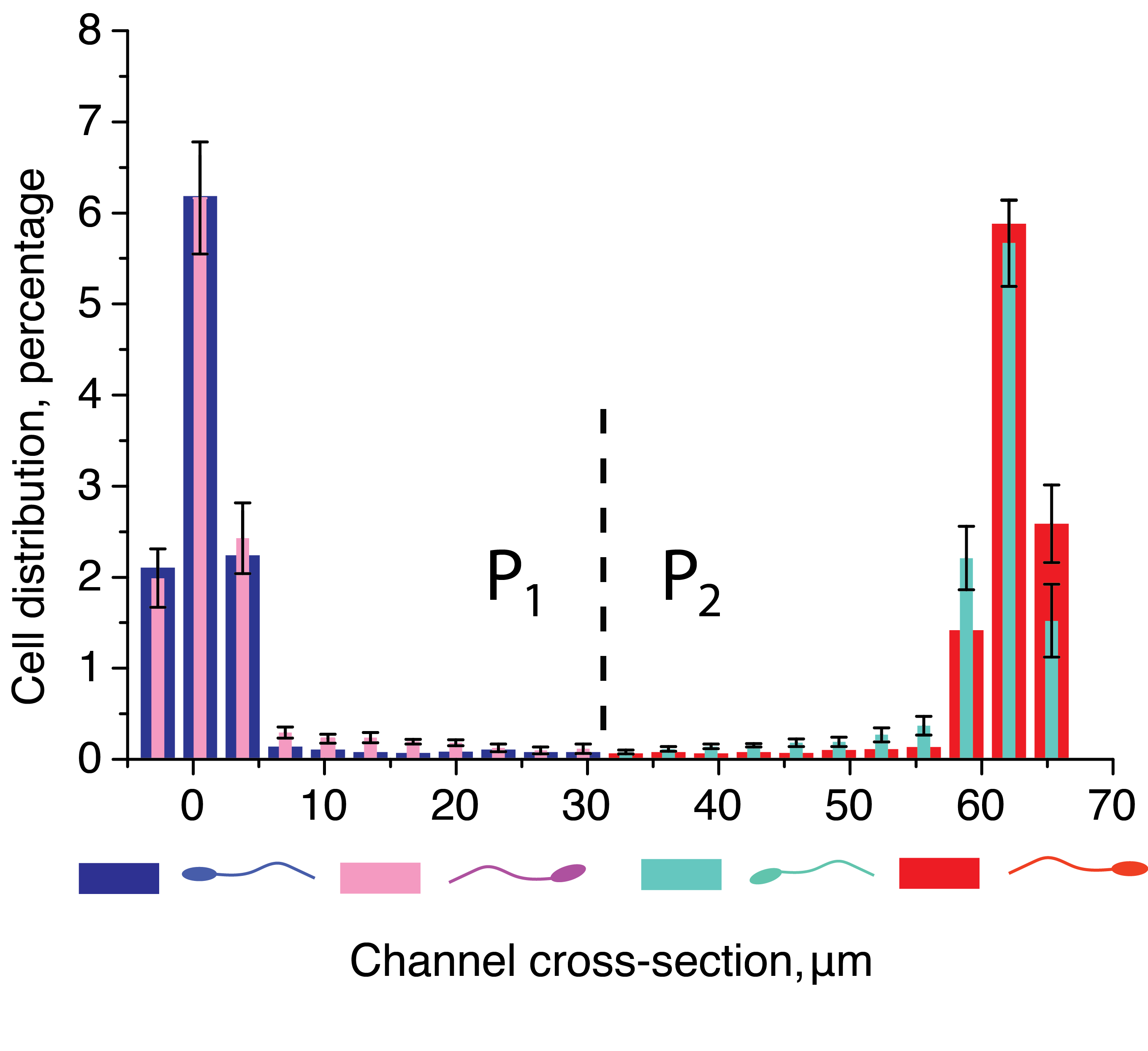 | b)  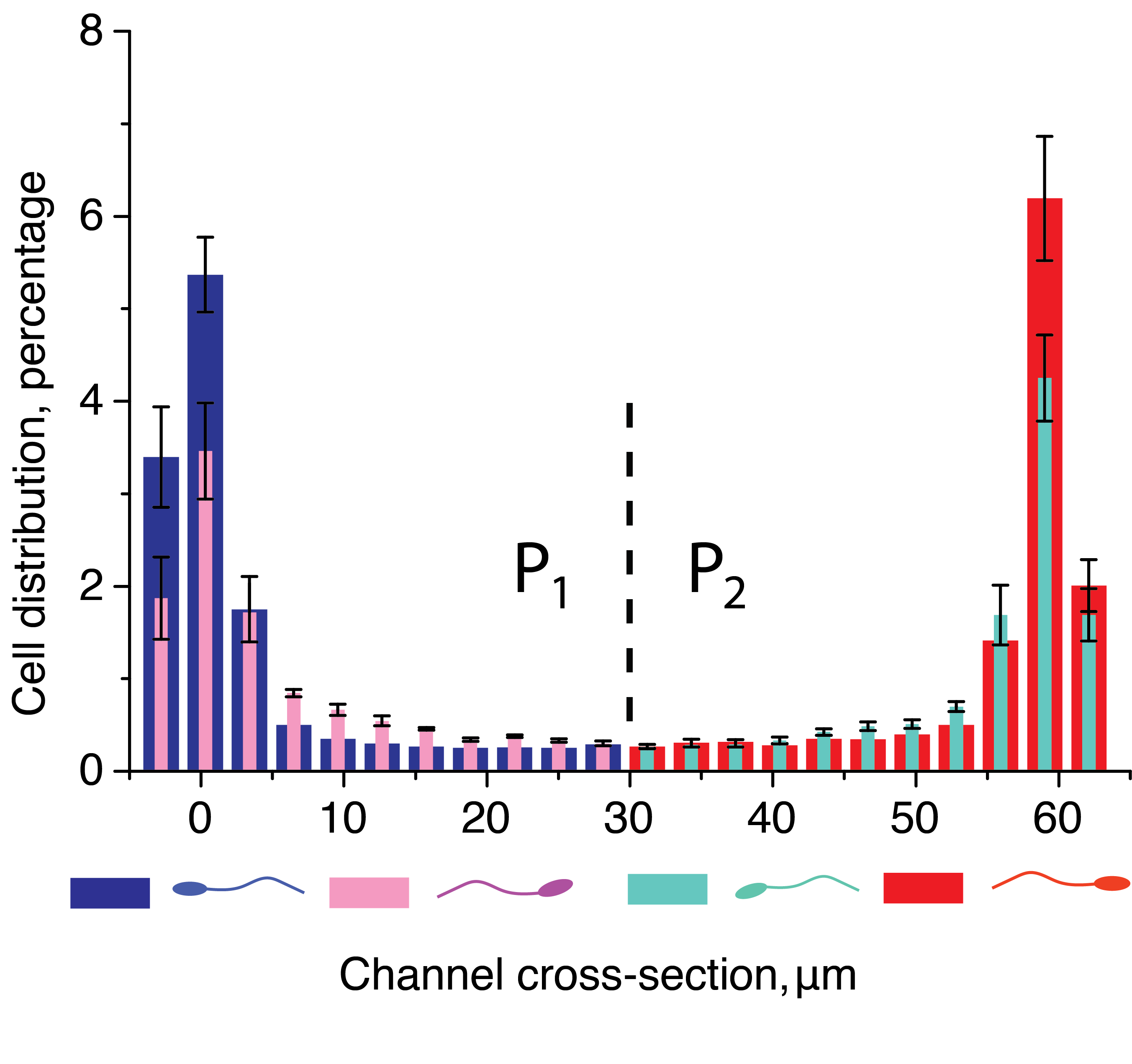 | | c)  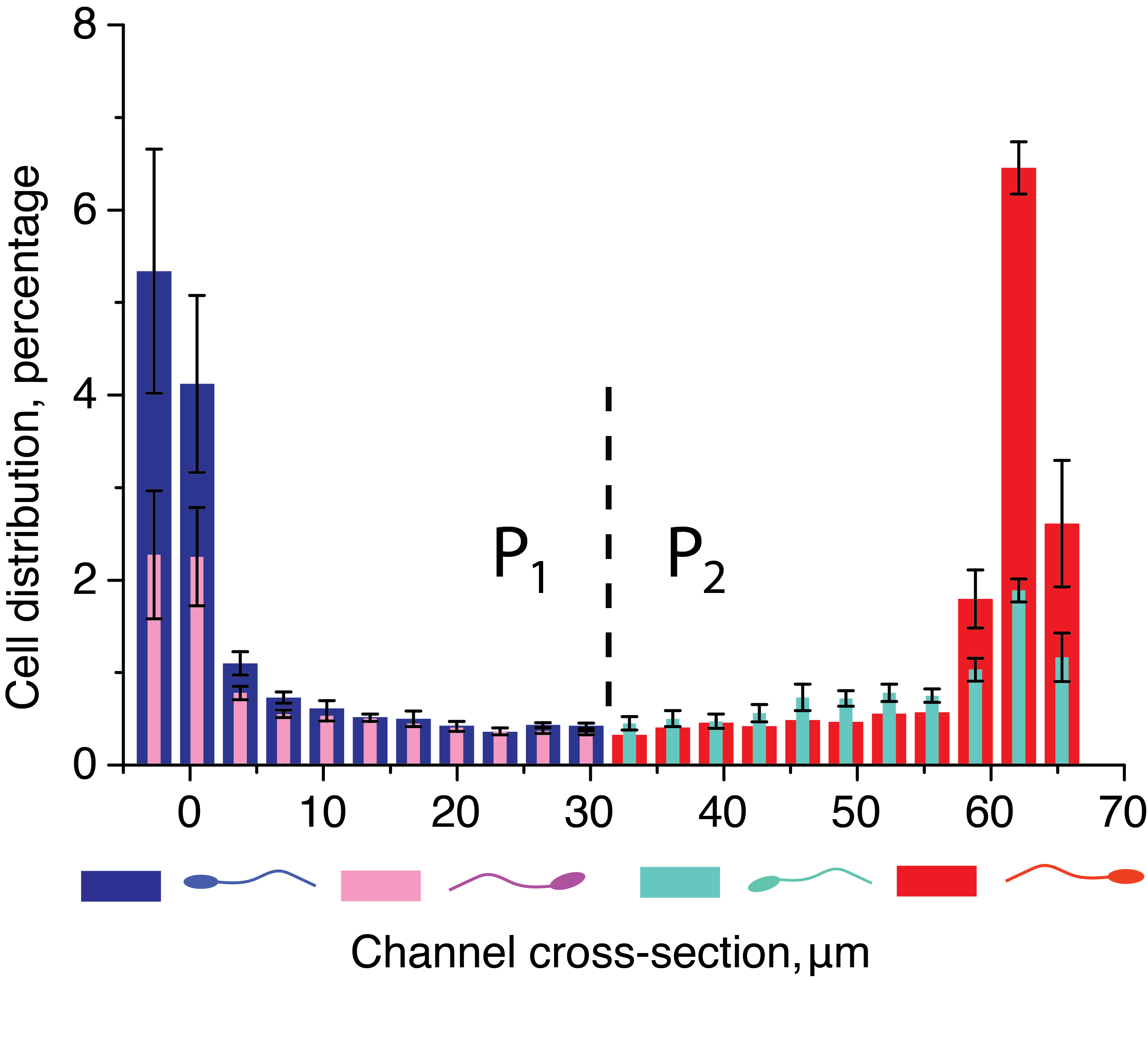 |
| --- | --- | --- | --- |
| d)  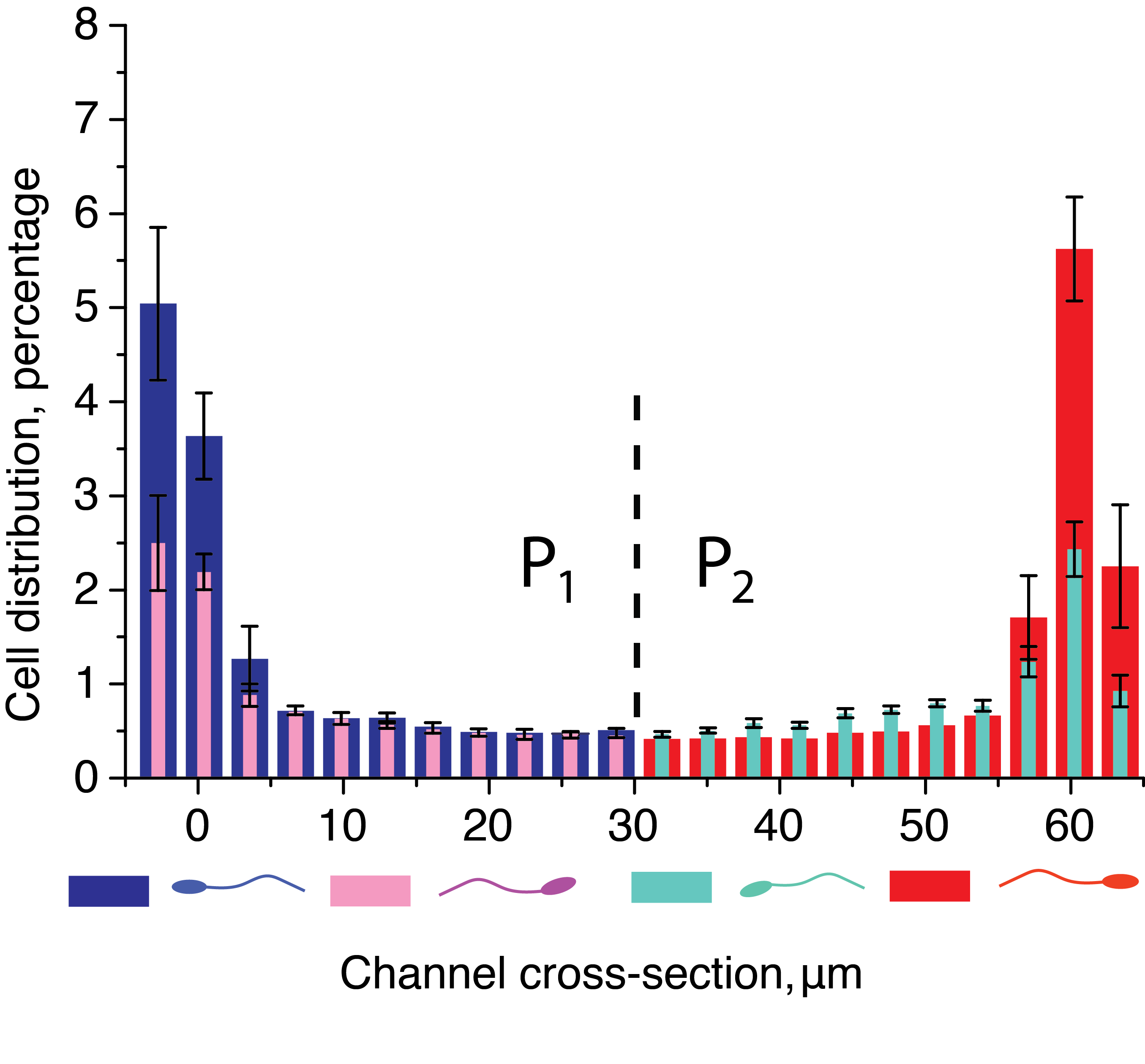 | | e)  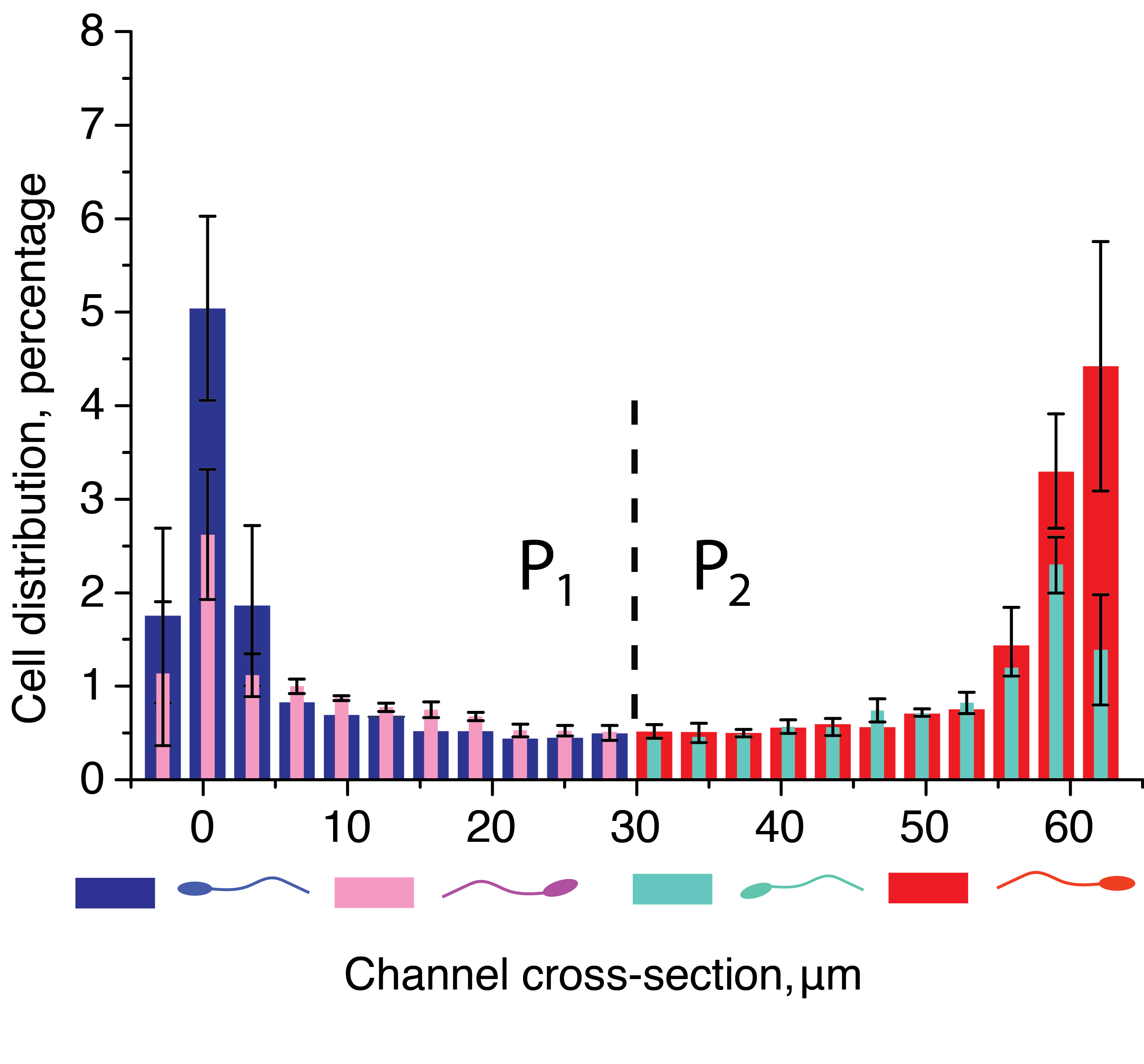 | |

Figure S3. Histograms of sperm cells average distribution across the channels at different concentrations: a) **n** ~ 0.4***n_0_**, 5 experiments; b) **n** ~ 1.1***n_0_**, 7 experiments; c) **n** ~ 2.5***n_0_**, 6 experiments; d) **n** ~ 3.2***n_0_**, 8 experiments; e) **n** ~ 3.9***n_0_**, 3 experiments. Colors of the bars indicate the swimming modes, error bars - standard errors.

a) b)


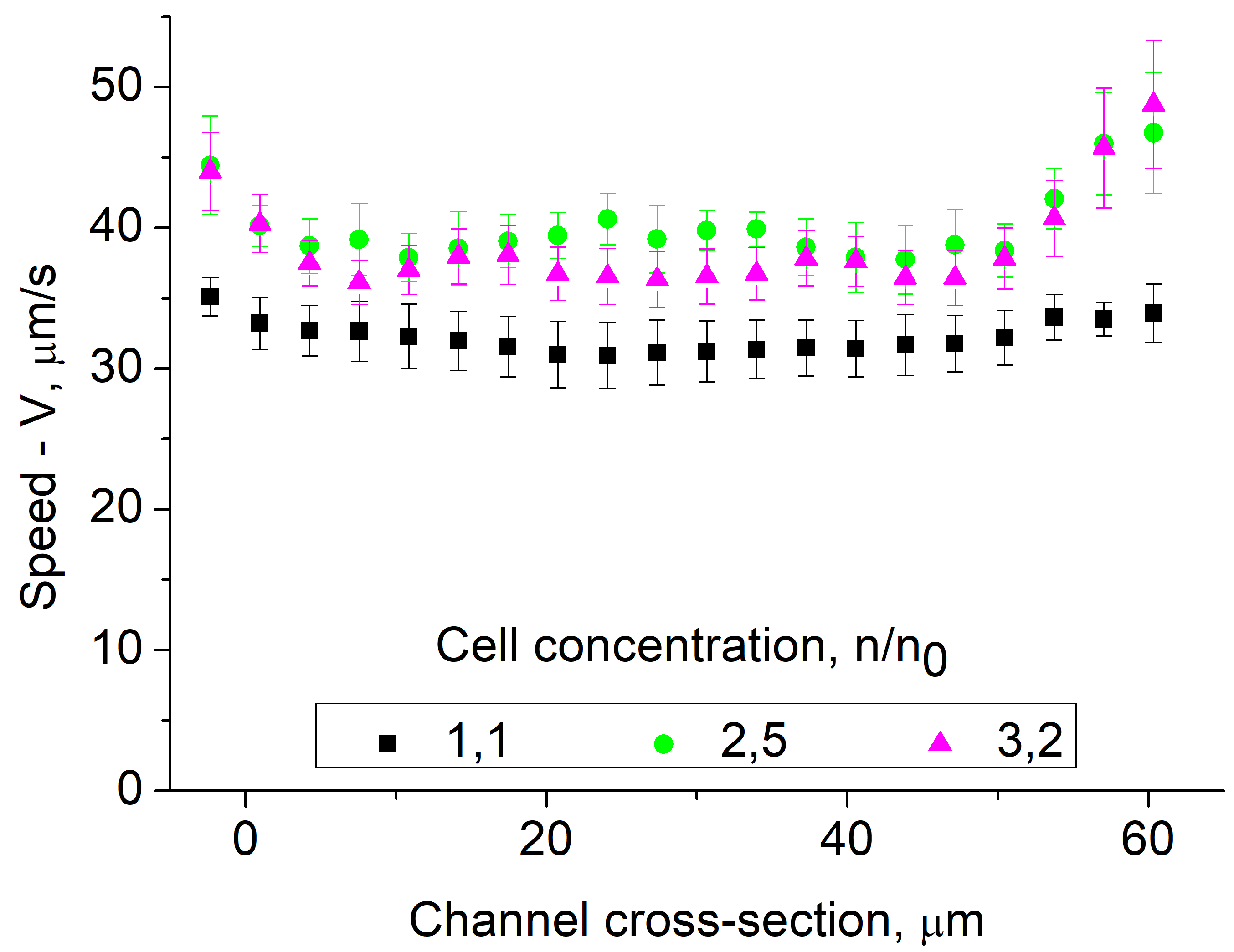

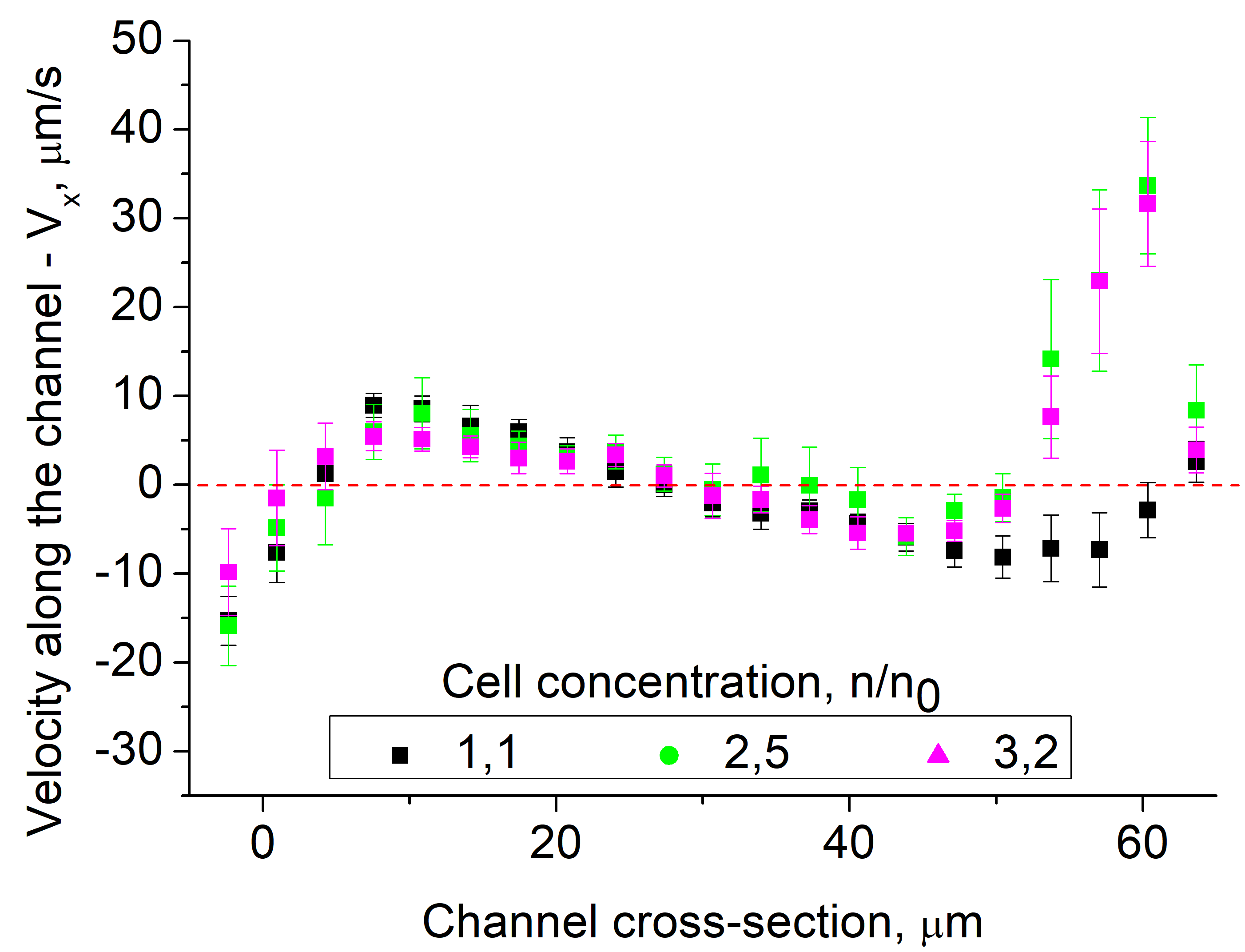


Figure S4. a) Spermatozoa average speed across the channel at different concentrations; b) average velocity along the channel axis at different concentrations.
